# Attachment Security Priming Delayed Negative Information-Related Attentional Disengagement Among Anxiously Attached Individuals: Evidence From Behavioral and Functional MRI Experiments

**DOI:** 10.3389/fpsyg.2022.913805

**Published:** 2022-06-09

**Authors:** Beiyi Wang, Xinyuan Peng, Fei Gao, Kaihua Zhang, Jianxin Zhang, Lili Wu

**Affiliations:** ^1^CAS Key Laboratory of Mental Health, Institute of Psychology, Chinese Academy of Sciences, Beijing, China; ^2^Department of Psychology, University of Chinese Academy of Sciences, Beijing, China; ^3^Department of Radiology, Shandong Provincial Hospital, Cheeloo College of Medicine, Shandong University, Jinan, China; ^4^Department of Radiology, Shandong Provincial Hospital Affiliated to Shandong First Medical University, Jinan, China; ^5^School of Psychology, Shandong Normal University, Jinan, China

**Keywords:** attachment security priming, attachment anxiety, attentional disengagement, dot-probe task, functional MRI

## Abstract

Although attachment security has been found to attenuate people’s experience of unpleasant information, how it modulates the attentional process toward such information remains unknown. The present study examined this issue by employing the dot-probe task in functional MRI. After completing the Experiences in Close Relationships-Revised questionnaire (ECR-R), 39 participants were asked to complete the dot-probe task in two conditions: the attachment security priming condition and neutral priming condition. The behavioral results revealed that individuals with high level of attachment anxiety exhibited larger attention disengagement from negative traits in the security priming condition than in the control condition. Correspondingly, the brain regions involved in attention regulation and shifting, such as the posterior cingulate and bilateral parietal area, were less activated among high anxiously attached individuals in the security priming condition. These results suggest a role of attachment security priming in regulating the emotional response in anxiously attached individuals during the attentional stage.

## Introduction

Attachment security is the feelings that one is worthy of being loved, that attachment figures are helpful when needed, and that the world is generally safe (Mikulincer and Shaver, 2007; Canterberry and Gillath, 2013). A temporally enhanced sense of attachment security has been found to benefit cognitive, emotional, and behavioral outcomes (Mikulincer and Shaver, 2007). One of the significant beneficial effects of attachment security is emotional regulation, or more specifically, mitigating the damage caused by various types of threats or unpleasant events (Karremans et al., 2011; Liao et al., 2017; Karreman et al., 2018; Oehler and Psouni, 2019; Wu et al., 2020). People that have higher attachment security report fewer negative experiences evoked by unpleasant stimuli (e.g., physical pain; Carnelley and Rowe, 2007; Master et al., 2009). In particular, the brain regions corresponding to the negative experience have been observed to show reduced activation among securely attached people than control participants (Coan et al., 2006, 2017; Eisenberger et al., 2011; Karremans et al., 2011; Norman et al., 2015). However, our understanding of how attachment security modulates the early process of threat information (i.e., attention) remains limited. To bridge this gap, the present study investigates the effect of attachment security on the information provided to cope with threats by focusing on the attentional process.

Attachment theory offers a privileged perspective to understand the different attention strategies adopted by people (Bowlby, 1982). According to the life span encompassing theoretical model, individuals with different internal working models of attachment process social information in a schematic manner congruent with their attachment representation (Dykas and Cassidy, 2011). Moreover, established working models of attachment shape the way in which people process social information by directing attention toward certain features and away from others in the early perceptual stages (Collins, 2003; Mikulincer and Shaver, 2003). The individual difference of attachment style was generally conceptualized in two basic dimensions—attachment avoidance and attachment anxiety (Brennan et al., 1998). Individuals with high attachment avoidance are uncomfortable with closeness and seek interpersonal distance and self-dependence (Mikulincer and Shaver, 2003). They appear to be less attentive to social and emotional events (Fraley et al., 2000). On the contrary, high anxiously attached individuals are motivated to seek acceptance or approval from others (Mikulincer and Shaver, 2003), and thus, they tend to selectively attend to information that can meet their goals (Collins, 2003). Compared with low anxious and low avoidance (i.e., secure) individuals, insecure attached individuals (high anxious and/or high avoidance), have been found to turn their attention away from negative words and images (Van Emmichoven et al., 2003; Dewitte et al., 2007a; Dewitte and De Houwer, 2008).

The temporally activated secure attachment internal working model aims to “*create a calm, warm, positive, and secure state of mind, which then contributes to a wide variety of outcomes*” (Mikulincer and Shaver, 2007, p. 201), including with regard to the attentional system. The activation of this model can lead insecure people to behave like secure ones. Previous studies have found that attachment security priming affects the attentional process by leading participants to be more open to negative information. For example, using the dot-probe task, Norman et al. (2015) found that participants in the attachment security priming group have larger attentional bias to negative stimuli and reduced activity in the amygdala than those in the control group. Wu et al. (2020) also found that attachment security priming leads participants to pay more attention to threatening images, as reflected by an enhanced P200, relative to the control priming group. Moreover, attachment security priming has been found to interact with people’s chronic attachment styles to affect how they process emotional information. Andriopoulou and Kafetsios (2015) found that secure priming turns high anxiously attached people’s attention toward positive emotional words compared with neutral priming. However, a recent study employing event-related potentials showed that supraliminal attachment security priming results in the increased allocation of attention toward negative information (vs. neutral information) among anxiously attached women, as reflected by an increased P300 (Ma et al., 2019). Although there are inconsistencies between the two studies by Andriopoulo and Ma, their results suggest that attachment security priming can lead to larger attentional bias to emotional stimuli among anxiously attached people.

These prior findings provide initial empirical evidence for the effect of attachment security priming on the attentional process. However, two issues remain to be addressed. The first issue concerns the specific components of attentional bias regulated by attachment security priming. Although previous studies have shown that attachment security priming enhanced selective attention to unpleasant stimuli, attentional bias could be due to the fast initial orienting to or slow disengagement from unpleasant stimuli, or both (Posner et al., 1987; Koster et al., 2004). Researchers have found that attentional orienting was affected by stimulus salience (Peng et al., 2017) and people’s motivation (Schultheiss and Hale, 2007), whereas attentional disengagement was affected by attention allocation or control (Ladouceur et al., 2009; Talbot et al., 2018). Therefore, differentiating orienting from disengagement would help unveil the regulating mechanism of attachment security on the attentional process.

The second issue is whether the effect of attachment security on the attentional process is valence-specific. Although it is established that attachment security priming can reduce the feelings elicited by negative and/or unpleasant information (Karremans et al., 2011; Liao et al., 2017; Karreman et al., 2018; Wu et al., 2020), one study has found that after the activation of secure schema, high anxiously attached individuals turn their attention to positive words compared with those in the control condition (Andriopoulou and Kafetsios, 2015). An individual’s attentional bias to emotional information also depends on their own attachment orientation as well as the valence conveyed by the information (Dewitte, 2011; Chavis and Kisley, 2012; Andriopoulou and Kafetsios, 2015). Therefore, including both positive and negative valence information would help us to determine the valence specificity of attachment security on the attentional process.

The present study investigated the effect of attachment security priming on attentional bias to negative information as well as the associated neural activation using functional MRI (fMRI). Regarding the first issue, we chose the dot-probe task to tap attentional bias into the two attentional components of attentional orienting and attentional disengagement. The former was indexed to the degree to which attention diverts away from the location of the emotion compared with neutral stimuli (Koster et al., 2004; Salemink et al., 2007; Tonta et al., 2019), while the latter was reflected by the extent to which attention shifted toward the location of the emotion compared with neutral stimuli (Salemink et al., 2007; Tonta et al., 2019). This task has been widely used in attention studies (Pishyar et al., 2008; Torrence and Troup, 2018; Wirth and Wentura, 2020; Yuan et al., 2021), including those have examined the effect of attachment orientation (Dewitte et al., 2007b; Dewitte and De Houwer, 2008) and attachment security priming (Norman et al., 2015). Meanwhile, neuroscientific techniques such as event-related potentials and fMRI can reliably unveil the neural underpinnings of attentional bias in dot-probe tasks (White et al., 2016; Torrence and Troup, 2018). The advantages of dot-probe tasks allow us to examine in depth how attachment security affects the attentional process. Regarding the second issue, we included both negative and positive trait words as stimuli to clarify whether attentional bias depended on the emotional information conveyed by those stimuli. As negative affect and positive affect are regarded as separate (Watson et al., 1999), we presented the negative and positive stimuli in separate blocks.

Regarding brain activation, we focused on the brain areas both associated with a sense of attachment security and related to the attentional process. First, Canterberry and Gillath (2013) showed that enhancing the sense of attachment security involves affective, cognitive, and behavioral components. Specifically, the sense of attachment security involves the (a) regions associated with positive affect, including the postcentral, temporal, and parietal areas; (b) regions regularly associated with cognitive control such as the prefrontal area; and (c) regions associated with motivation and the anticipation of outcomes, including the supplementary motor area and supramarginal gyrus. Further, the brain activations associated with processing valence information conveyed by negative words were also considered. Norman et al. (2015) examined the effect of attachment security priming on emotional regulation using the dot-probe task and found that it attenuated the activation in the left amygdala evoked by negative words. Therefore, in the present study, we also focused on the activation in this area to examine the effect of attachment security priming.

The present study also focused on the brain regions associated with attentional orienting and shifting/reallocation. The orienting process involved the frontal eye field (Petersen and Posner, 2012). The allocation of attention required the contributions of multiple brain regions, including the cingulate gyrus, parietal lobe, and prefrontal cortex (Small et al., 2003; Fan et al., 2005; Torrence and Troup, 2018). Other areas also involved in the attention to emotional stimuli (Torrence and Troup, 2018), such as the anterior cingulate, anterior insula, and inferior frontal gyrus were additionally considered. Compared with neutral information, negative information increases brain activation in these regions.

Hence, the present study examined whether attachment security priming enlarges attentional bias to negative information, especially among anxiously attached people. We proposed two main hypotheses. Specifically, H1 stated that attachment security priming would evoke brain areas related to affectional and cognitive processes and attenuate negative-related neural activation. H2 stated that attachment security priming would make people more open to negative information compared with the control priming, especially among those with high attachment anxiety. In addition, as Andriopoulou and Kafetsios (2015) found that enhanced attachment security among individuals with anxious attachment leads them to pay more attention to positive words in the attachment priming condition, we also investigated the effect of attachment security priming on attention to positive information. Individuals with high attachment anxiety may pay more attention to positive information in the attachment security priming condition than in the control condition. As trait anxiety has been found to be related to attachment anxiety (Mikulincer and Shaver, 2003), in the present study, we also needed to assess this index and control it in data analyses to rule out alternative explanations, such as the modification in attention bias to negative information is due to trait anxiety rather than attachment anxiety that we intended to examine.

## Materials and Methods

### Participants

The participants included 39 undergraduate or graduate Chinese students (19 women, 18–26 years old, mean age = 23.03 years, SD = 1.61). All the participants were in long-term stable romantic relationships of more than 6 months (range = 7–72 months, *M* = 21.72 months, *SD* = 15.23). They were all right-handed and their visual or corrected visual acuity was normal. They were free of any history of neurological or psychiatric problems. All participants were recruited through a post on line and those who met the eligibility criteria (e.g., age and right-handed) voluntarily contact us. The Institutional Review Board of the Institute of Psychology at the Chinese Academy of Sciences approved the experimental protocol. The participants were informed that participation was voluntary. They also provided informed consent and received the corresponding remuneration after the experiment. The data from one additional participant (female) were not included in the analysis because they were incomplete during the fMRI recording.

The sample size was determined based on two principles: (i) the number of participants was estimated by using G*Power 3.1 in F-tests ANOVA, repeated measures for within factor and within-between interactions. With *α* = 0.05, power (1-β) = 0.8, and a medium effect size *f* = 0.25, the sample size was calculated to be 24, which was the largest among the F-tests to detect both the main effect of priming and the interaction between priming and valence and (ii) the number of participants usually involved in prior fMRI studies using within-participant design in the attachment-related area (e.g., Bartels and Zeki, 2004, *N* = 20; Coan et al., 2006, *N* = 16; Eisenberger et al., 2011, *N* = 21; Karremans et al., 2011, *N* = 15); regarding the specific focus on attention among those with a high level of attachment anxiety, the number of participants in previous neuroscientific studies that examine moderating effect attachment anxiety was taken as the criteria (e.g., Gillath et al., 2005, *N* = 20; Ma et al., 2019, 20 anxiously attached participants and 19 securely attached participants).

### Measures

#### Attachment Styles

The attachment styles of the participants were measured using the Experiences in Close Relationships-Revised questionnaire (ECR-R; Fraley et al., 2000). This measurement has two subscales measuring attachment anxiety (e.g., “I am afraid that I will lose my partner’s love”) and attachment avoidance (e.g., “I find it difficult to allow myself to depend on romantic partners”). Items are scored on a seven-point Likert scale where 1 = not at all and 7 = very much. For each subscale, there are 18 items (negatively worded items are reverse-scored), and the responses are averaged to obtain a subscale score. A higher score indicates higher attachment anxiety or attachment avoidance. In the present study, the Cronbach’s alpha was 0.92 and 0.87 for attachment anxiety and attachment avoidance, respectively.

#### Anxiety

Anxiety was assessed using the State–Trait Anxiety Inventory (STAI) subscale (Spielberger et al., 1983) that assesses trait anxiety, that is, how people “generally feel” about anxiety. It consists of 20 items, each of which is scored on a four-point Likert scale where 1 = not at all and 4 = very much so, resulting in total scores from 20 to 80. In this study, Cronbach’s alpha was 0.92.

### Attachment Security Priming Task

In the attachment security priming condition, the participants were instructed to recall and write down a personal experience containing an attachment security scheme (Bartz and Lydon, 2004). The instruction for this priming was as follows: “*Please recall a personal experience in which you faced a problem that you could not solve on your own and made you feel depressed. People who are close with you such as your parents or your friends are always sensitive and responsive to your distress. You turn to them for help. They help you to solve the problem and make you comfortable. They want to help you only because they love you and set aside other activities to assist you.*” After recalling this personal event, the participants wrote down a short description of it and their feelings about it.

In the neutral priming group, they were instructed to recall and write down a neutral, emotionally irrelevant personal experience (Wu et al., 2020). The instruction for this priming was as follows: “*Please recall a personal experience of going to the store and buying some daily necessities. You stood in front of the shelf and compared some brands, and finally chose one commodity.*” The participants wrote down the location of the grocery store and names of several daily necessities they might buy there.

### Stimuli Material

We selected 18 positive and 18 negative trait adjectives from the likability ratings of 555 personality trait words (Anderson, 1968). A likability rating of 313 was considered as the cutoff, which represents the center or neutral point in the word list. The rating differences between the negative adjectives and the cutoff and the positive adjectives and the cutoff were not significant (*M_positive-cutoff_* = 202.17 ± 15.96, *M_negative-cutoff_* = 197.67 ± 21.85; *t* = 0.71, *p* = 0.49, *d* = 0.24). In total, 108 neutral words were selected from the Chinese Affective Words System (Wang et al., 2008) as neutral stimuli. These traits constructed 18 positive/neutral word pairings (e.g., “honest/glass”), 18 negative/neutral word pairings (e.g., “cowardly/ant”), and 36 neutral/neutral word pairings (e.g., “computer/echo”).

### Procedure

First, the participants completed the ECR-R and STAI scales online. After a week, they came to the fMRI laboratory. Each of the participants was asked to complete both the attachment security and the control priming tasks outside the scanner. After that, they performed a dot-probe task. fMRI scans were acquired during the dot-probe task performance.

Before the dot-probe task, a simple instruction was presented on the screen to lead the participants to recall the experience they had written outside the scanner. The short instruction for attachment security priming was as follows: “*Please now recall the experience and feelings that you wrote outside the scanner about getting help from a close person.”* The instruction for control priming was as follows: “*Please recall the experience and feelings that you wrote outside the scanner about going to the store and buying some daily necessities.*”

The negative stimuli (negative/neutral word pairings) and positive stimuli (positive/neutral word pairings) were presented in separate blocks. The formal experiment was conducted with four types of blocks, namely, the attachment security priming/negative block (security/negative), attachment security priming/positive block (security/positive), neutral priming/negative block (neutral/negative), and neutral priming/positive block (neutral/positive). Each participant completed all four types of blocks and we counterbalanced the order of the priming and trait word valence between the participants.

Each of the two positive blocks (security/positive and neutral/positive) contained 18 positive/neutral word pairings and 18 neutral/neutral word pairings which were randomly selected from the 36 neutral/neutral word pairings. Each of the two negative blocks (security/negative and neutral/negative) contained 18 negative/neutral word parings and the other 18 neutral/neutral word pairings. In each block, each pair of words was presented twice, in which the positions of the two words were switched. Finally, positive blocks contained 36 positive/neutral word pairings and 36 neutral/neutral word pairings, whereas negative blocks contained 36 negative/neutral word pairings and 36 neutral/neutral word pairings.

Each block consisted of 144 trials, including 36 emotional congruent trials, 36 emotional incongruent trials, and 72 neutral trials. In the emotional congruent trial, the probe replaced the emotional word, while the neutral word was replaced by the probe in the emotional incongruent trial. In the neutral trials, the probe replaced one of the two words in the neutral/neutral word pairing. The order of the presentation of each trial was randomized.

Each trial began with a 500 ms fixation cross (“+”) followed with the word pair to the left and right on the screen for 500 ms. After the disappearance of the two words, the dot probe replaced one of the two words for about 2000 ms. Participants were instructed to indicate the location of the dot probe by pressing the left or right buttons. The dot disappeared after the button had been pressed, and a fixation appeared until the beginning of the next trial. Each trial lasted 2000 ms or 4,000 ms. The location difference was balanced in the left and right fields of vision.

## Data Analysis

### Behavioral Data Analysis

Behavioral statistical analyses were performed in SPSS for Windows (version 20.0; IBM Corp., Armonk, NY, United States). Trials with response latencies less than 200 ms or longer than 1,000 ms as well as trials for wrong reactions were excluded as outliers (0.69–5.56% in each condition, security/positive: *M* = 1.00%; SD = 1.40%; security/negative: *M* = 0.71%, SD = 0.99%; neutral/positive: *M* = 1.02%; SD = 1.47%; and neutral/negative: *M* = 0.86%; SD = 1.28%).

Regarding the reaction time and accuracy, we performed two repeated ANOVAs of 2 (priming group: attachment security priming vs. control priming) × 2 (congruency: congruent vs. incongruent) × 2 (word valence: positive vs. negative).

Furthermore, to examine the specific attentional process involved in the dot-probe task, the following three attentional components were calculated as:

1. *Attentional bias* = RT*_emotional incongruent trials_* – RT*_emotional congruent trials_*
2. *Attentional orienting* = RT*_neutral trials_* – RT*_emotional congruent trials_*
3. *Attentional disengagement* = RT*_emotional incongruent trials_* – RT*_neutral trials_*

The positive score of attentional bias indicated that individuals produced an attentional bias for emotional stimuli. The higher score of attentional orienting indicated a greater speed to pay attention to the emotional stimuli (i.e., higher scores = rapid orienting). The higher score for attentional disengagement indicated difficulty disengaging attention from the emotional stimuli (i.e., higher scores = delayed disengagement). These three components of attention were analyzed using three 2 (priming group: attachment security priming vs. control priming) × 2 (word valence: positive vs. negative) repeated ANOVAs. In addition, to examine the potential interaction effects between attachment priming and attachment styles on the three attentional components, we performed three 2 (priming group: attachment security priming vs. control priming) × 2 (word valence: positive vs. negative) ANCOVAs, using the scores of attachment anxiety, attachment avoidance, and trait anxiety as the covariates.

### fMRI Data Acquisition and Analysis

All the participants were scanned with a 3.0 T scanner (Philips “Achieva” TX, Best, the Netherlands) using an eight-channel phased-array head coil. A participant’s head was fixed with foam pads to minimize their head movements throughout the experiment. The structural data were acquired using the T1-weighted 3D turbo field echo sequence: repetition time (TR) = 8.1 ms, echo time (TE) = 3.7 ms, slice thickness = 1 mm, field of view = 24 × 24 cm^2^, and voxel size = 1 × 1 × 1 mm^3^. For the functional imaging, the whole-brain coverage of 40 axial slices was acquired using a T2*-weighted echo-planar imaging sequence based on the blood oxygenation level-dependent contrast (TR = 2000 ms, TE = 35 ms, image matrix = 64 × 64, slice thickness = 4 mm, field of view = 200 mm × 200 mm, voxel size = 3.1 mm × 3.1 mm × 4.0 mm, flip angle = 90°).

Image preprocessing and statistical analysis were performed using SPM12 software.^1^ The first five functional EPI volumes of each session were discarded to allow for T1 stabilization. All the remaining functional EPI images were slice-time corrected, realigned for head motion correction, coregistration with individual structural images, segmented for normalization to a Montreal Neurological Institute template and resampled to create 3.5-mm isotropic voxels, and spatially smoothed using a Gaussian filter with 8-mm full width half maximum.

For the model, the onset times of each prime/probe were modeled as single events with durations of zero. Six realignment parameters were included to account for movement-related variability. A high-pass filter with a cutoff frequency of 1/128 Hz was used to correct for low-frequency components and serial correlation correction using an autoregressive AR(1) model.

Contrasts were created for each participant, and these were then entered into the second level for the group analyses. Four parametric contrast images corresponding to the four experimental conditions (attachment security priming/positive, attachment security priming/negative, neutral priming/positive, and neutral priming/negative) were generated at the individual level. These were submitted to a two-factor ANOVA with priming (attachment security priming vs. neutral priming) and valence (positive vs. negative) as the two within-participant factors at the second level for all the participants using a random effect model.

### ROI Analysis

Owing to the focus on the activation in the left amygdala, we conducted our analyses using anatomically defined ROIs. The ROI was created using WFU-Pickatlas.^2^ Parametric estimation values were extracted from this ROI using Marsbar, a toolbox that provides routines for SPM that allow researchers to perform ROI analyses (v0.44, Brett et al., 2002).^3^ The activation in the left amygdala was extracted from each participant’s first-level maps for the four contrasts (security priming/positive, security priming/negative, neutral priming/positive, and neutral priming/negative) and submitted to a two-factor ANOVA with priming (attachment security priming vs. neutral priming) and valence (positive vs. negative) as the two within-participant factors.

### Regression Analyses

Regression analyses were used to examine the associations between the signal intensity in the brain regions and scores on the two attachment style dimensions. We focused of the neural basis that reflected the attentional process in the dot-probe task and created four parametric contrast images correspondingly (attachment security priming/positive > attachment security priming/neutral, attachment security priming/negative > attachment security priming/neutral, neutral priming/positive > neutral priming/neutral, and neutral priming/negative > neutral priming/neutral). When assessing the statistical relations between the brain regions, we focused on those brain areas related to the attentional process, including the cingulate gyrus, parietal lobe, prefrontal area, anterior insula, and amygdala.

The brain areas reported were significant at *p* < 0.001 (voxel level, uncorrected) for the 10 contiguous voxels for the main contrasts and for the contrasts with the covariates, unless we specifically point out.

## Results

The results are presented in four sections. In the first, we provide the descriptive statistics of the sample characteristics, and then, the results of the factor analysis of general behavioral performance including accuracy and reaction time. Next, we presented the results of the factor analysis of the three attentional indices. In the third section, we present the fMRI results associated with attachment priming, valence, and their interaction as well as the ROI result in the left amygdala, which would reveal the effect of attachment security priming. Finally, we presented the regression results that reflect the associations between the signal intensity in the brain regions and scores of the two attachment style dimensions in the control and security priming conditions, respectively.

### Sample Characteristics

In our final sample, the participants’ average attachment anxiety score was 3.16 (SD = 1.12) and the average attachment avoidance score was 2.49 (SD = 0.75). In addition, the average trait anxiety score was 39.51 (SD = 8.11, range = 20–59).

### Behavioral Results

#### Accuracy

The repeated ANOVA results revealed a marginally significant congruency effect (*F* = 3.64, *p* = 0.06, $\eta_{p}^{2}$ = 0.09), such that the participants responded more accurately in the congruent trials (*M* = 0.988, *SD* = 0.003) than in the incongruent trials (*M* = 0.985, *SD* = 0.003). Neither the main effect of the attachment priming condition (*F* = 0.08, *p* = 0.78, $\eta_{p}^{2}$ = 0.002) nor the main effect of the word valence condition (*F* = 0.40, *p* = 0.53, $\eta_{p}^{2}$ = 0.01) was significant. Interestingly, the interaction between word valence and congruency reached significance (*F* = 4.46, *p* = 0.04, $\eta_{p}^{2}$ = 0.11). To further explain this interaction, we conducted two 2 (priming: attachment security priming vs. control priming) × 2 (congruency: congruent vs. incongruent) ANOVAs separately for the positive and negative words. For the positive words, we found a significant congruency effect (*F* = 6.94, *p* = 0.012, $\eta_{p}^{2}$ = 0.15), indicating that the participants responded more accurately in the congruent trials (*M* = 0.99, *SD* = 0.003) than in the incongruent trials (*M* = 0.98, *SD* = 0.004). This effect was not found for the negative words (*Fs* < 0.39, *ps* > 0.55).

#### Reaction Time

The repeated ANOVA results revealed a marginally significant main effect of priming (*F* = 3.59, *p* = 0.07, $\eta_{p}^{2}$ = 0.086). Specifically, compared with the control priming condition (*M* = 399.57, SD = 6.80), the participants responded faster in the attachment security priming condition (*M* = 390.54, SD = 6.56). Further, there was a marginal effect of congruency (*F* = 3.17, *p* = 0.08, $\eta_{p}^{2}$ = 0.08), with the participants responding slightly faster in the congruent trials (*M* = 393.27, SD = 6.46) than in the incongruent trials (*M* = 396.83, SD = 6.18). The main effect of word valence was not significant (*F* = 0.12, *p* = 0.73, $\eta_{p}^{2}$ = 0.003). All the interaction effects failed to reach significance (*Fs* < 2.61, *ps*>0.11). Furthermore, regarding the three indices of attentional bias, neither significant main effects (*Fs*<2.61, *ps*>0.11) nor significant interaction effects (*Fs*<0.48, *ps*>0.49) were observed.

#### The Effect of Attachment Styles on Attentional Bias

The ANCOVA results revealed significant effects for only attentional disengagement. There was a marginally significant interaction between attachment priming and attachment anxiety (*F* = 3.84, *p* = 0.06, $\eta_{p}^{2}$ = 0.10) and a significant interaction between word valence and attachment anxiety (*F* = 4.91, *p* = 0.03, $\eta_{p}^{2}$ = 0.12). None of the other main effects and interactions reached significance for attentional disengagement (*Fs*<2.63, *ps*>0.11; For the attentional bias and attentional orienting results, see the first part of the Supplementary Material).

To interrogate these effects, we performed another two ANCOVAs to examine attentional disengagement from the negative or positive word probes separately. Attachment anxiety, attachment avoidance, and trait anxiety were included as the covariates. Further, to better explain the interaction between attachment priming and attachment styles, we excluded trait anxiety and performed another two ANCOVAs on attentional disengagement.

For the negative word probes, the ANCOVA for attentional disengagement (with attachment anxiety, attachment avoidance, and trait anxiety as the covariates) yielded a significant interaction between priming and attachment anxiety (*F* = 6.78, *p* = 0.01, $\eta_{p}^{2}$ = 0.16). The simple slope analyses revealed that attachment anxiety was significantly associated with attentional disengagement in the attachment security priming condition (*β* = 0.37, *t* = 2.43, *p* = 0.02), which was consistent with our hypothesis that those with high attachment anxiety would pay more attention to negative information after attachment security priming. By contrast, in the control condition, the association between attachment anxiety and disengagement was not significant (*β* = −0.18, *t* = −1.14, *p* = 0.26). No other significant effects were observed (*Fs*<2.68, *ps*>0.11).

For the positive probes, the ANCOVA results for attentional disengagement (with attachment anxiety, attachment avoidance, and trait anxiety as the covariates) showed the significant main effect of attachment anxiety (*F* = 5.85, *p* = 0.02, $\eta_{p}^{2}$ = 0.14). To identify the direction of the attachment anxiety effect, we performed a hierarchical regression analysis for attentional disengagement in the positive condition, using attachment anxiety as an independent variable and the mean value of attentional disengagement as a dependent variable in both the attachment security priming condition and the control priming condition. The results revealed a significant negative association between attachment anxiety and attentional disengagement from the positive words (*β* = −0.40, *t* = −2.69, *p* = 0.01), which was contrary to the final hypothesis that supposed individuals with high level attachment anxiety may pay more attention to positive information in the attachment security priming condition than in the control condition. No other significant main effects or interaction effects were observed (*Fs*<0.70, *ps*>0.41).

When controlling without trait anxiety, however, the pattern of the results remained unchanged (see the second part of the Supplementary Material).

### Neuroimaging Results

#### Priming Effect

To assess the effect of attachment security priming, we determined from the fMRI data which brain areas were more activated in the attachment priming condition than in the neutral priming condition (collapsed across the positive and negative blocks; see Table 1).

**Table 1:** Activations associated with attachment security priming (Attachment priming > neutral priming).

Brain Regions^a^	Side	MNI coordinates			*t* values	Z scores	Cluster size
		X	Y	Z			
Superior Frontal Gyrus Frontal_Sup_Medial_L	L	−16	54	2	2.77	2.73	14
Sub-gyral temporal lobe	L	−36	−2	−28	3.11	3.05	15
Sub-gyral occipital and temporal lobe	L	−32	−66	−4	2.85	2.81	10

a the threshold was set at *p* < 0.005, uncorrected.

#### Trait Valence Effect

The positive versus negative trait words contrast revealed that processing negative words (relative to positive words) was associated with significant activation in the postcentral gyrus and superior temporal gyrus. No significant activation was observed when processing positive words relative to negative words (see Table 2).

**Table 2:** Activations associated with valence (Negative traits > positive traits).

Brain Regions	Side	MNI coordinates			*t* values	Z scores	Cluster size
		X	Y	Z			
Postcentral Gyrus Parietal_Sup_L	L	−26	−36	54	3.89	3.79	34
Superior Temporal Gyrus Temporal_Sup_L	L	−48	0	−6	3.56	3.48	17

#### Priming × Emotional Valence Interaction

To explore the neural underpinnings supporting the effect of attachment security priming on negative information processing, we located the brain regions sensitive to the interaction between priming and emotional valence (see Table 3).

**Table 3:** Activations associated with the interaction between priming and valence processing.

Brain Regions	Side	MNI coordinates			*t* values	Z scores	Cluster size
		X	Y	Z			
*(AP-AN)-(CP-CN)*							
Inferior Frontal Gyrus OFCpost_R Insula_R	R	38	20	−18	3.53	3.46	16
*(AN-AP)-(CN-CP)*							
Inferior Frontal Gyrus Frontal_Inf_Tri_R Frontal_Inf_Oper_R	R	60	22	14	3.93	3.83	43
Cingulate Gyrus	R	18	2	40	3.43	3.36	16
Inferior Parietal Lobule Angular_L	L	−40	−58	42	3.42	3.35	19
*AN>CN*^a^							
Inferior Frontal Gyrus^a^ Frontal_Inf_Tri_R Frontal_Inf_Oper_R	R	60	20	14	3.64	3.56	21

AP, Attachment_positive; AN, attachment_negative; CP, control_positive; and CN, control_negative. No significant activations were observed in the contrast of AP > CP, CP > AP, and CN > AN at the threshold of *p* < 0.001, voxels > 10.

a For the examination of simple effect, the threshold was set at p < 0.0005, voxels > 10, uncorrected.

To discern the activation pattern in the inferior frontal gyrus and right ventral anterior insula, the activation from this region was extracted from a spherical ROI (centered on the maxima coordinates: *x* = 38, *y* = 20, *z* = −18; radius = 5 mm) and submitted into a two-way ANOVA with priming (security priming vs. control priming) and valence (positive vs. negative) as the within-participant factors. The results showed that the priming effect approached significance, *F* = 3.51, *p* = 0.069, $\eta_{p}^{2}$ = 0.084, indicating lower activation in the attachment security priming condition than in the control priming condition. Moreover, a significant interaction was observed, *F* = 14.93, *p* < 0.001, $\eta_{p}^{2}$ = 0.282.

**Figure 1:**
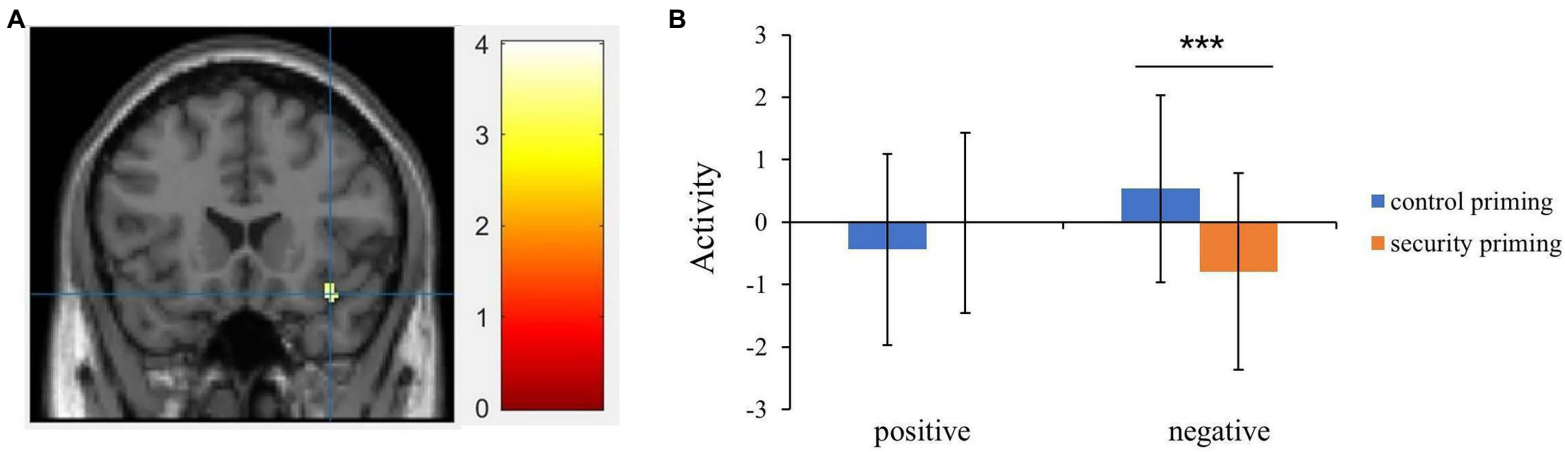
**(A)** The interaction between priming (attachment security priming vs. control priming) and valence (positive vs. negative) showed activation in right inferior frontal gyrus (extended to insula). **(B)** Extracted beta values from inferior frontal gyrus showed the activation pattern between control priming and attachment security priming conditions. Error bars indicate the standard deviation of means (^***^p < 0.001).

Further to interpret the interaction, the paired-sample *t* test results revealed that in the control condition, the activation in this region evoked by negative words (*M* = 0.53, SD = 1.50) was larger than that after attachment security priming (*M* = −0.79, SD = 1.58, *t* = 4.18, *p* < 0.001, *d* = 0.67). However, for positive words, no significant difference was observed between control priming (*M* = −0.44, *SD* = 1.53) and attachment security priming (*M* = −0.01, SD = 1.44, *t* = −1.25, *p* = 0.22, *d* = −0.20; Figure 1). Finally, the valence effect was not significant, *F* = 0.30, *p* =. 59.

#### Activation in the Left Amygdala

The activation in the left amygdala was submitted into a two-way ANOVA with priming (security priming vs. control priming) and valence (positive vs. negative) as the within-participant factors. The results showed a significant priming effect, *F* = 5.11, *p* = 0.03, $\eta_{p}^{2}$ = 0.118, indicating lower activation in attachment security priming than in control priming. No other significant effect emerged, *F*s < 2.44, *p*s >. 12.

**Figure 2:**
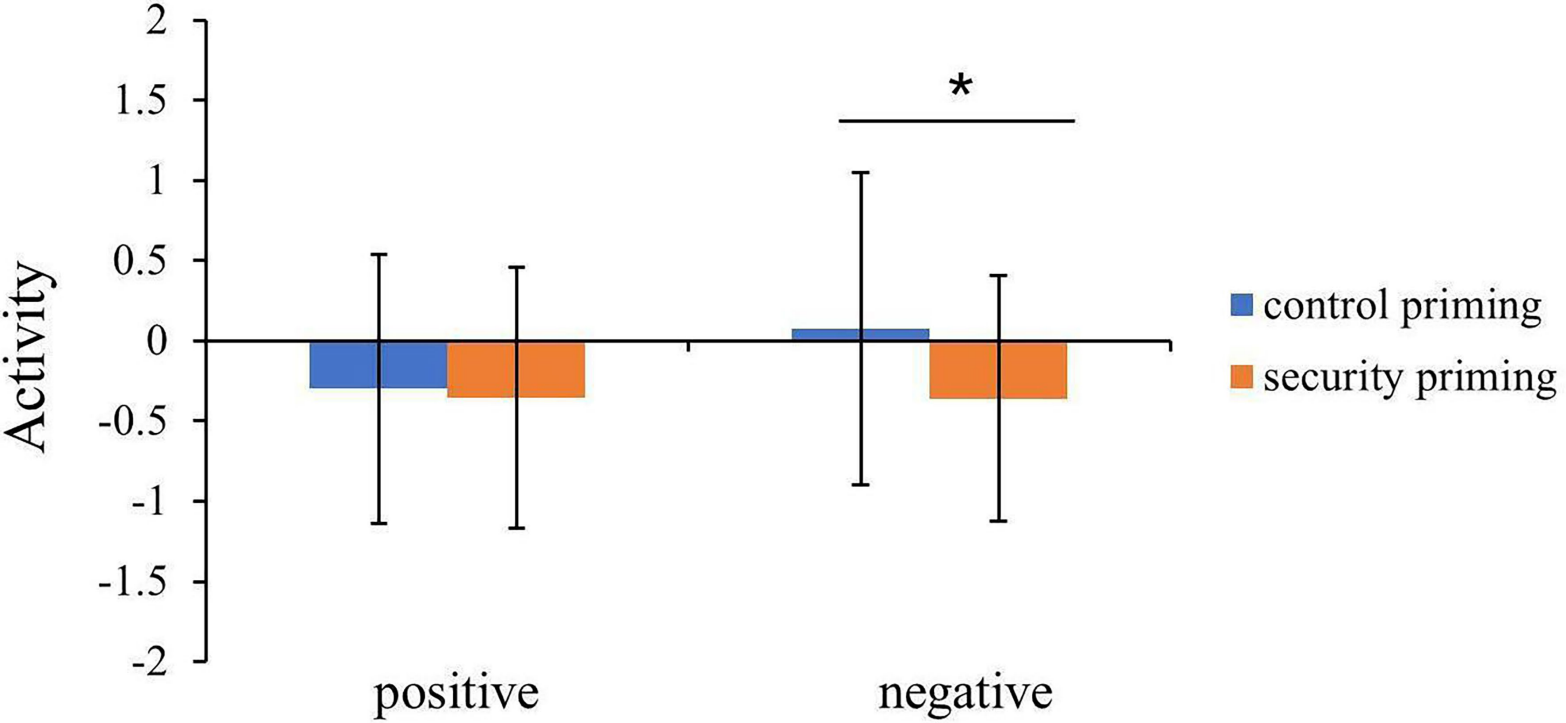
Extracted beta values from left amygdala showed the activation pattern between control priming and attachment security priming conditions. Error bars indicate the standard deviation of means (^*^p < 0.05).

To interrogate these effects, we conducted additional comparisons for the positive and negative words separately to determine whether one of these two types was driving the priming effect. The activation in the amygdala evoked by the positive words under security priming and control priming was not significantly different, *t* = 0.32, *p* = 0.76. However, the activation in the amygdala evoked by negative words in the control condition (*M* = 0.08, SD = 0.97) was larger than that in the security priming condition (*M* = −0.36, *SD* = 0.77, *t* = 2.56, *p* = 0.01, *d* = 0.38; Figure 2).

#### Correlations With Attachment Anxiety

Following the analysis of attentional disengagement from the negative stimuli that revealed a moderating effect of attachment anxiety on the priming effect of attachment security, we hypothesized that high anxious participants have a different pattern of activation during attention to negative stimuli than low anxious ones do. To test this prediction, we explored whether attachment anxiety is associated with the activation pattern detected in the whole-brain comparisons of the negative/neutral pair with the neutral/neutral pair.

We first regressed the negative/neutral pair–neutral/neutral pair contrast on attachment anxiety in the control and attachment security priming conditions. The analysis revealed that in the control condition, high anxious attachment was associated with decreased activation mainly in the medial frontal area, limbic system, and bilateral brainstem. By contrast, in the attachment security priming condition, high anxious attachment was associated with decreased activation in the posterior cingulate cortex, bilateral inferior parietal gyrus, and bilateral brainstem (see Table 4). Attachment avoidance and trait anxiety were also modeled in the two regression models in order to control their effects on brain activation.

**Table 4:** Correlations with attachment anxiety.

Brain Regions	Side	MNI coordinates			*t* values	Z scores	Cluster size
		X	Y	Z			
Control priming							
*Decreased activation*							
Culmen Right Cerebellum Cerebellum Anterior Lobe Cerebellum_6_R	R	22	54	26	3.77	3.43	21
Right Cerebrum Sub-lobar Extra-Nuclear Hippocampus_R Thalamus Thal_LGN_R Lateral Geniculum Body Limbic Lobe Hippocampus_R Parahippocampa Gyrus	R	26	−23	−4	4.35	3.86	49
Sub-lobar Left Cerebrum Left Brainstem Thal_VPL_L Midbrain Extra-Nuclear Thalamus Thal_LGN_L	L	−20	−22	−4	4.28	3.81	38
Left Cerebrum Parahippocampa Gyrus Limbic Lobe ParaHippocampal_L	L	−18	−38	−4	3.83	3.48	11
Right Cerebrum Parahippocampa Gyrus Limbic Lobe Sub-lobar Precuneus_R	R	16	−38	2	4.13	3.70	18
Sub-lobar Right Cerebrum Extra-Nuclear Insula Insula_R	R	26	−20	12	3.85	3.49	39
Right Cerebrum Rolandic_Oper_R Insula Sub-lobar Parietal Lobe Inferior Parietal Lobule Temporal_Sup_R	R	48	−32	22	4.39	3.89	28
Inferior Parietal Lobule Left Cerebrum Parietal Lobe SupraMarginal_L	L	−46	−42	26	4.22	3.77	25
Cingulate Gyrus Right Cerebrum Cingulate_Mid_R Limbic Lobe Frontal Lobe Cingulate_Mid_L Supp_Motor_Area_L Frontal_Sup_2_R Frontal_Sup_Medial_L Superior Frontal Gyrus Supp_Motor_Area_R	L/R	14	18	40	5.05	4.35	276
Frontal Lobe Left Cerebrum Frontal_Sup_Medial_L Superior Frontal Gyrus	L	−8	28	52	3.94	3.56	15
Attachment security priming							
*Decreased activation*							
Right Brainstem Pons Midbrain Left Brainstem Culmen Right Cerebellum Cerebellum Anterior Lobe Cerebellum_3_R	R/L	−6	−26	−30	4.86	4.22	166
Parietal Lobe Precuneus	L	−16	−52	36	3.71	3.38	10
Cingulate Gyrus Limbic Lobe Right Cerebrum Cingulate_Mid_R	R	4	−42	40	3.62	3.31	16
Inferior Parietal Lobule Parietal Lobe Left Cerebrum Parietal_Inf_L	L	−52	−60	44	3.88	3.51	20
Inferior Parietal Lobule Parietal Lobe Right Cerebrum Parietal_Inf_R Angular_R	R	52	−60	48	3.50	3.22	12

No significant increased activations were observed in control condition and attachment security priming condition at the threshold of *p* < 0.001, voxels > 10.

## Discussion

The present study was aimed to investigate how attachment security priming affects attentional process to negative information. In supporting of H1, we found that attachment security priming selectively activated the brain area related to the affectional and cognitive processes, consistent with previous findings (Canterberry and Gillath, 2013). It should be note that in reporting the main effect of attachment security priming, we used a relative liberal threshold (*p* < 0.005, voxel >10), which was following previous attachment-related studies (Gillath et al., 2005; Canterberry and Gillath, 2013). The attenuated the negative activation in the left amygdala and right ventral insula after attachment security priming as compared to control priming was in line with previous findings of Norman et al. (2015), which further supported the effect of attachment security priming. On the other hand, attachment security priming led to slow attentional disengagement from negative information among high anxiously attached participants, and the neural results also showed that in the attachment security priming condition, high anxious attachment was associated with attenuated activation in the posterior cingulate cortex and bilateral inferior parietal gyrus, which has been related with attention shifting in prior studies (e.g., Fan et al., 2005; Torrence and Troup, 2018). These results supported H2 that attachment security priming would make people more open to negative information, especially among individuals with high attachment anxiety.

Previous behavioral studies have observed that high anxiously attached people direct their attention away from negative information (Dewitte et al., 2007b; Dewitte and De Houwer, 2008). Although two studies have reported enhanced P3 (Mark et al., 2012) and LPP (Zilber et al., 2007) with anxious attachment, these results might be affected by trait anxiety. According to attention theory, the attentional process is affected by top-down and bottom-up regulation. The turning away of attention from negative information among high anxiously attached individuals reflects top-down regulation. After attachment security priming, we observed the opposite results, that is, high anxiously attached individuals showed slow disengagement from negative information. This implied that the activation of secure attachment schema made those individuals open to negative information and did not avoid it by diverting their attention away from it. The result is consistent with Ma et al.’s (2019) finding that attachment security priming enhances high anxiously attached women’s attention to infant pictures with negative expressions. At the same time, the effect of attachment security priming was valence-specific. That is, the priming effect emerges only when attention is paid to negative information, not positive information.

The presented results suggested that the effect of attachment priming benefits high anxiously attached individuals but not secure individuals or high avoidantly attached individuals. These findings are also accordant with those of Ma et al. (2019). The lack of evident of this effect among high avoidantly attached individuals may be due to the supraliminal priming approach used in our study. Bryant and Chan (2017) proposed that individuals with high attachment avoidance could inhibit the activation of secure attachment representation, which make them less vulnerable to the supraliminal priming manipulation. Previous studies also showed that subliminal security priming is more effective for individuals with high attachment avoidance (e.g., Ma et al., 2019).

In the attachment security priming condition, attachment anxiety is associated with attenuated activation in the posterior cingulate cortex and bilateral inferior parietal area. The posterior cingulate cortex and bilateral parietal area have been found to be related to attention shifting (Small et al., 2003; Fan et al., 2005; Torrence and Troup, 2018). The high activation in these regions reflected the attention redirecting process. Compared with low anxiously attached participants, high anxiously attached ones exhibited reduced activation in these areas, suggesting less attention shifting or slow disengagement from negative information.

Diverting attention from negative information is an effective way to regulate one’s emotion and could reduce one’s experiences associated with negative stimuli (Frankenstein et al., 2001; Dolcos et al., 2020). Younger et al. (2010) showed that the mechanism of attachment security priming on relieving pain is distinct from that of distracting attention. The presented findings showed that attachment security priming delayed a high anxiously attached participant’s attentional disengagement from negative information. This raises the question of whether the slower disengagement from negative stimuli led to stronger negative experiences. To test this possibility, we examined the correlations between attentional disengagement and the activation in the ventral anterior insula and left amygdala evoked by negative stimuli in the attachment security priming condition but found no significant positive associations (with the insula, *r* = −0.22, *p* = 0.18; with the amygdala, *r* = −0.12, *p* = 0.45; as a comparison, in the neutral priming condition, with the insula, *r* = 0.01, *p* = 0.96; with the amygdala, *r* = 0.16, *p* = 0.33). These results ruled out the possibility that slow disengagement from negative information led to elevated experience.

Highly anxious attachment was associated with quick disengagement from positive traits in the present study. This result was inconsistent with the finding of Andriopoulou and Kafetsios (2015), which showed that anxiously attached people had larger attentional bias to positive information in the attachment security priming condition than in the control condition. One possible interpretation for this contrasting finding is the difference in positive stimuli used in the present study and by Andriopoulou and Kafetsios (2015), who used positive emotion words (e.g., rejoice, optimism) as positive stimuli, whereas we used trait words. According to the self/other model of attachment, anxiously attached people have a negative model of self, which is inconsistent with positive traits. Therefore, they tend to disengage their attention from such information. Nevertheless, further research is warranted to determine the relationship between attachment anxiety and attention to positive information.

We point out three main limitations of our study. First, to avoid group differences in attachment styles and the other dimensions at the baseline, we adopted a within-participant design. Such within-participants design has also been showed to be effective in examining the effect of attachment security (Bartz and Lydon, 2004; Coan et al., 2006; Beauregard et al., 2009; Master et al., 2009; Eisenberger et al., 2011; Karremans et al., 2011). However, Gillath et al. (2008) pointed out that attachment security priming may have a long-term effect. In view of this, the effects of control priming may be contaminated by attachment security priming. Correspondingly, the within-participant design could weaken the difference between the two priming effects. To remove the potential impact of the long-term effects of attachment security priming, future work could use a between-participant design to explore the relationship between attachment security and attention.

Second, we did not use a manipulation check to verify the effectiveness of priming. On the one hand, the effectiveness of the supraliminally administered priming procedure has been established by previous studies (Bartz and Lydon, 2004; Mikulincer and Shaver, 2007; Mikulincer et al., 2011; Wu et al., 2020; for a review, see Gillath and Karantzas, 2019, also see a recent meta-analysis, Gillath et al., 2022). On the other hand, despite the common usage of manipulate check in psychological research in assessing the effectiveness of a treatment, some researchers argued that it would cause some problems, such as motivating the participants to guess the researcher’s hypothesis, changing physiological responses, and changing the thinking way and task performance (Hauser and Schwarz, 2015; Fayant et al., 2017; Hauser et al., 2018). Considering the specific within-participant design, if we used manipulation checks to assess participants’ sense of security in both the control and the attachment security conditions, they might guess the intention of this study, which may affect the results (Hauser et al., 2018). Nevertheless, as compared with the control condition, the brain activity in left amygdala was decreased under attachment security priming condition, which was consistent with previous study (Norman et al., 2015), reflecting the effectiveness of the priming procedure.

Finally, although the dot-probe task has been widely used to assess attentional bias (e.g., Dewitte et al., 2007b; Dewitte and De Houwer, 2008; Pishyar et al., 2008; Norman et al., 2015; Wirth and Wentura, 2020; Yuan et al., 2021; Fernandes-Magalhaes et al., 2022), its reliability remains in controversial (Torrence and Troup, 2018; Chapman et al., 2019). Several methodological factors, such as has stimulus onset asynchrony (Chapman et al., 2019), stimuli characteristic (e.g., emotional facial expressions; Torrence and Troup, 2018), and participants characteristics (Van Rooijen et al., 2017), have been assumed to account for the differences observed in studies used dot-probe task. Regarding this issue, some researchers suggested to utilize neuroscientific technique when using dot-probe task to measure attention process (White et al., 2016; Torrence and Troup, 2018). Other researchers also purposed that it would be useful to include the neutral baseline condition (neutral—neutral trials) when using the dot-probe task to explore the attentional mechanism (Fernandes-Magalhaes et al., 2022). Nevertheless, as Torrence and Troup (2018) indicated, although the dot-probe task has extended our knowledge of attentional bias, a new task that can reliably assess attention bias, engagement, and disengagement is necessary. The use of such a reliable attentional task would be helpful to further examine the conclusions of the present study.

In summary, the present study determined the effect of attachment security priming on the attentional process as well as its underlying neural mechanism. The results showed that attachment security priming leads to a slow attentional disengagement from negative information among participants with high attachment anxiety. Correspondingly, the brain regions involved in attention regulation and shifting, such as the posterior cingulate and bilateral parietal area, were less activated among high anxiously attached individuals in the security priming condition. These results indicated that attachment anxiety modulates early cognitive process (e.g., attentional process), and future studies should consider to include relevant measures of individual differences when exploring the cognitive effect of attachment security priming.

## Data Availability Statement

The raw data supporting the conclusions of this article will be made available by the authors, without undue reservation.

## Ethics Statement

The studies involving human participants were reviewed and approved by the Institutional Review Board of the Institute of Psychology at the Chinese Academy of Sciences. The patients/participants provided their written informed consent to participate in this study.

## Author Contributions

BW and LW conceived and designed the experiments, analyzed the data, and wrote the manuscript. BW performed the experiments. XP, FG, KZ, and JZ revised the manuscript. All authors contributed to the article and approved the submitted version.

## Funding

This work was supported by the National Natural Science Foundation of China (grant no. 31871125).

## Conflict of Interest

The authors declare that the research was conducted in the absence of any commercial or financial relationships that could be construed as a potential conflict of interest.

## Publisher’s Note

All claims expressed in this article are solely those of the authors and do not necessarily represent those of their affiliated organizations, or those of the publisher, the editors and the reviewers. Any product that may be evaluated in this article, or claim that may be made by its manufacturer, is not guaranteed or endorsed by the publisher.

## Footnotes

^1^ https://www.fil.ion.ucl.ac.uk/spm/software/spm12/

^2^ https://www.nitrc.org/projects/wfu_pickatlas

^3^ http://marsbar.sourceforge.net
