# Supplementary material for: Attachment Security Priming Delayed Negative Information-Related Attentional Disengagement Among Anxiously Attached Individuals: Evidence From Behavioral and Functional MRI Experiments
Source: Front Psychol. 2022 Jun 9;13:913805. doi: 10.3389/fpsyg.2022.913805 (PMC9218902; doi:10.3389/fpsyg.2022.913805)
Supplement: Supplementary file 1 [file Table_1.DOCX]

Supplementary Material

# The results of covariance analysis of attention bias and attention orienting

Regarding the attentional bias, neither the significant main effects were observed (*Fs*＜0.86, *ps*＞.36), nor the significant interaction effects were observed (*Fs*＜1.52, *ps*＞.23).

Regarding the attentional orienting, neither the significant main effects were observed (*Fs*＜0.99, *ps*＞.33), nor the significant interaction effects were observed (*Fs*＜1.14, *ps*＞.29).

# Interaction between priming and attachment style without controlling trait anxiety

In order to better explain the interaction only between attachment priming and attachment styles, we excluded trait anxiety and performed another ANCOVA on attentional disengagement in separately positive and negative condition, using priming condition as a within participants’ factor. Only attachment anxiety and attachment avoidance were included as covariates.

In the positive condition, the ANCOVA on attentional disengagement yielded a significant main effect of attachment anxiety (*F* = 6.21, *p* = 0.02, *η_p_^2^* = 0.15). To further explain the main effect of attachment anxiety, we performed a hierarchical regression analysis for attentional disengagement in positive condition, using attachment anxiety as independent variable, and the mean value of attentional disengagement in attachment security priming condition and it in control priming condition as dependent variable. The result revealed a significant negative association between attachment anxiety and attentional disengagement in positive condition (*β*= - 0.40, *t* = -2.69, *p* = .01), which indicating that individuals with higher attachment anxiety had faster speed to disengage from positive stimuli. No other significant main effects or interaction effects were observed (*Fs*＜1.32, *ps*＞.26).

In the negative condition, the ANCOVA on attentional disengagement yielded a significant interaction between prime and attachment anxiety (*F* = 6.69, *p* = 0.01, *η_p_^2^* = 0.16). The simple slope analyses revealed attachment anxiety was significantly associated with attentional disengagement in the attachment security priming condition (*β*= .37, *t* = 2.43, *p* = .02), nor in the control condition (*β*= -.18, *t* = -1.14, *p* = .26), indicating that individuals with higher attachment anxiety showed slower speed to disengage from negative stimuli under attachment security priming. No other significant effects were observed (*Fs*＜2.89, *ps*＞.10).

In general, the results of controlling and not controlling trait anxiety are the same mode.
